# In the Era of Advanced Microsurgery, Is There Still a Place for Pedicled Abdominal Flaps? A Retrospective Analysis

**DOI:** 10.3390/jcm14051696

**Published:** 2025-03-03

**Authors:** Marta Jagosz, Piotr Węgrzyn, Maja Smorąg, Patryk Ostrowski, Michał Bonczar, Michał Chęciński, Szymon Manasterski, Ahmed Elsaftawy

**Affiliations:** 1 Department of Plastic and Hand Surgery, St. Jadwiga Śląska Hospital, 55-100 Trzebnica, Poland; 2 Department of Anatomy, Jagiellonian University Medical College, 31-008 Kraków, Poland; 3 Youthoria, Youth Research Organization, 31-008 Kraków, Poland

**Keywords:** abdominal flaps, upper limb, reconstruction, injury, trauma

## Abstract

**Background**: Upper extremity reconstruction poses a significant challenge due to the complex anatomical and functional requirements of the hand and forearm. While free flaps have become the gold standard, pedicled abdominal flaps remain a valuable alternative, especially in cases where microsurgical anastomosis is contraindicated or unfeasible. This study evaluates the efficacy and outcomes of free-style pedicled abdominal flaps in reconstructing complex upper limb defects. **Methods**: A retrospective review was conducted on 20 patients who underwent soft tissue reconstruction of the upper extremity using free-style pedicled abdominal flaps between January 2019 and June 2024. Patient demographics, injury mechanisms, flap design, postoperative complications, and clinical outcomes were analyzed. Flap design was customized to defect size and location, utilizing single, double, triple, and tunneled flap configurations where necessary. **Results**: Stable soft tissue coverage was achieved in all cases without the need for additional free flap procedures. Complications included surgical site infections (n = 2), marginal necrosis (n = 2), partial flap necrosis (n = 2), and complete necrosis (n = 2), with no cases requiring free flap conversion. Long-term follow-up revealed no finger stiffness or loss of hand function. Donor site morbidity was minimal, with only one case requiring a split-thickness skin graft for closure. **Conclusions**: Despite advancements in microsurgical techniques, free-style pedicled abdominal flaps remain a vital reconstructive option for upper limb defects, particularly in patients with complex, large, or circumferential injuries. Their versatility, reliable vascularity, and ability to conform to various defect shapes underscore their enduring relevance in modern reconstructive surgery.

## 1. Introduction

The upper extremity possesses distinctive anatomical features and functional demands, making its reconstruction a complex and highly specialized endeavor. Optimal soft tissue coverage should prioritize the restoration of function, facilitate early mobilization, reestablish sensory feedback, and achieve aesthetic harmony. Equally important is ensuring a durable repair while minimizing morbidity at the donor site.

During the 1970s and 1980s, pedicled flaps from the groin and abdomen were the primary approach for reconstructing hand and forearm defects, with numerous large-scale studies supporting their use [1,2]. Despite the rising usage of free flaps in recent decades, favored for their ability to avoid cumbersome positioning, reduce hospital stays, and enable earlier rehabilitation, pedicled flaps remain a valuable option in specific cases. Indications for their use include electric burns, pediatric patients under two years old, and situations requiring future vascularized toe transfers, amongst others [3]. The groin flap, first described by McGregor and Jackson in 1972 [2], continues to be utilized due to its simplicity, rapid harvest, and thin donor site, which is especially advantageous in obese patients. Unlike free flaps, groin flaps avoid the risk of vascular compromise from end-to-side anastomoses and do not require the same high level of microsurgical expertise, making them a viable option in resource-limited settings [4,5].

On the other hand, pedicled flaps from the groin and abdomen are associated with several significant drawbacks. The abdominal pedicled flap, in particular, often requires a two-stage procedure, which is frequently followed by the need for debulking surgeries. This approach not only prolongs hospitalization but also delays the initiation of physiotherapy for the injured hand. Such delays increase the risk of joint stiffness, especially at the level of the metacarpophalangeal joints, which can significantly impact functional recovery.

The aim of this study is to present our approach for soft tissue coverage of the upper extremity, highlighting the design and incision of free-style pedicled abdominal flaps. We discuss the benefits of this technique over conventional methods and its potential advantages, drawing on our experience with 20 cases. In each case, the free-style pedicled abdominal flap was selected as the most feasible treatment option, taking into account the patient’s overall condition and specific clinical needs.

## 2. Materials and Methods

We retrospectively analyzed a case series of 20 consecutive patients who underwent upper extremity soft tissue reconstruction using free-style pedicled abdominal flaps in our department between January 2019 and June 2024. The study included only patients for whom free flaps were not a viable treatment option due to their poor health condition and for whom primary treatment with skin grafts was deemed inappropriate. More specifically, the inclusion criteria encompassed patients with extensive injuries involving potential recipient vessels, rendering microsurgical anastomosis for free flap reconstruction unfeasible. Additionally, patients with large or circumferential soft tissue defects that precluded the use of local or pedicled flaps for adequate coverage were included. High-risk surgical scenarios, such as extensive burns or necrotizing infections where microsurgical anastomosis carried a high risk of failure, also met the inclusion criteria. On the other hand, patients were excluded if their upper extremity defects could be adequately treated using local flaps or free tissue transfer without contraindications. Likewise, those with small or superficial defects that could successfully be managed with skin grafting alone were not included in the study. Other exclusion criteria included extensive previous scarring of the donor site and impaired general health conditions, such as severe vascular disease. The cohort consisted of 3 female and 17 male patients, ranging in age from 9 to 82 years. The mechanisms of tissue damage included industrial-type injuries, tissue necrosis in 4 patients, high-voltage electrical burns in 3 cases, and 1 case of thermal burn injury. Clinical data were obtained from the hospital’s database and patient records. Informed consent was obtained from all patients, adhering to the ethical principles outlined in The Code of Ethics of the World Medical Association (Declaration of Helsinki) for research involving human participants.

Preoperative marking is performed according to the size and location of the defect, independent of the well-documented perforator sites in the abdominal region. The nature and extent of tissue loss dictate the flap design. Unlike the classical abdominal flap, the length-to-breadth ratio of the free-style pedicled abdominal flap is significantly larger, often three to four times greater. Regarding flap design, the length-to-breadth ratio was determined based primarily on the unique anatomical and clinical requirements of the defect coverage in each case. The larger ratio compared to classical abdominal flaps allowed for greater versatility and sufficient reach, essential for addressing multiple defects on an upper limb simultaneously. To ensure adequate coverage, the flap length is designed to be at least as large as the size of the defect requiring reconstruction. When appropriate, the use of adjacent but separate flaps is considered. In addition to single flaps, double- and triple-pedicled abdominal flaps were employed in selected cases. Tunneled flaps were utilized to achieve comprehensive coverage for circumferential-like soft tissue defects. In cases involving multiple defects, multiple small abdominal flaps were designed and applied to address the specific needs of each defect. This versatile approach enables the reconstruction of complex defects while maintaining the integrity of the donor site.

Following the customized flap design, elevation commenced within the superficial fat layer and progressed beneath the superficial fascia. Flaps were raised to a sufficient length to ensure adequate coverage of the defect. The affected limb was then positioned against the abdominal donor site to assess flap suitability and confirm proper alignment. When inserting the flap into the defect, suturing began from the posterior edge, advancing towards the anterior side. To improve the cosmetic outcome and reduce tension during closure, the fat at the skin edge of the flap was trimmed in a beveled manner. This technique not only enhanced the aesthetic result but also contributed to the prevention of marginal flap necrosis. In most cases, flap fixation was achieved using 3-0 polypropylene or nylon mattress sutures, or alternatively, a surgical stapler was employed for efficient and secure closure.

Effective management of the donor site is a critical aspect of the procedure. A pinch test is performed preoperatively to evaluate the feasibility of primary closure and to guide the design of the flap for optimal defect coverage. If primary closure of the donor site cannot be achieved without excessive tension, a thin skin graft is used to close the area. In certain cases, secondary aesthetic refinement of the abdominal scar, including abdominoplasty, may be considered as part of a staged approach.

Postoperative care focuses on maintaining patient comfort and facilitating early physiotherapy. Dressings are applied in a manner that supports mobility while protecting the flap. Uninjured digits are left unbound to preserve the functional positioning of the hand. To prevent skin irritation, gauze is placed between the upper limb and the abdominal skin, as well as in the axillary region. Dressing changes are performed every 1 to 2 days, ensuring proper hygiene and optimal conditions for healing.

## 3. Results

Soft tissue defect coverage was achieved using a variety of free-style pedicled abdominal flaps, each tailored to the specific characteristics of the defect. The following flap configurations were utilized: 11 cases of single-pedicled abdominal flaps, 2 cases of double-pedicled flaps, 1 case of a triple-pedicled flap, 4 cases of tunneled abdominal flaps, and 5 individual pedicled flaps for each finger in a single patient. Additionally, one case involved the use of a pedicled abdominal flap combined with a non-vascularized bone graft harvested from the iliac crest.

**Figure 1:**
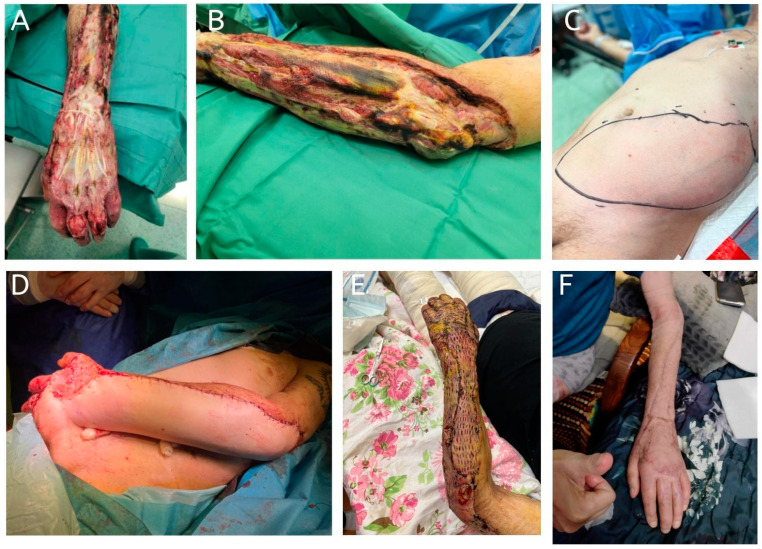
A 64-year-old patient with a history of chronic myeloid leukemia presented with a complete circumferential upper extremity defect following debridement for necrotizing fasciitis. Due to complete flap failure, flap division was performed on day 11. The failed flap was maintained as a “biological dressing”, promoting granulation tissue formation and facilitating wound closure with a split-thickness skin graft 10 days later. (**A**,**B**): Preoperative upper limb; (**C**): extended abdominal flap design marked on the patient’s abdomen; (**D**): inset of the pedicled abdominal flap into the defect; (**E**): application of a split-thickness skin graft over the granulated wound; (**F**): final result showing the reconstructed upper extremity 5 years postoperatively.

**Figure 2:**
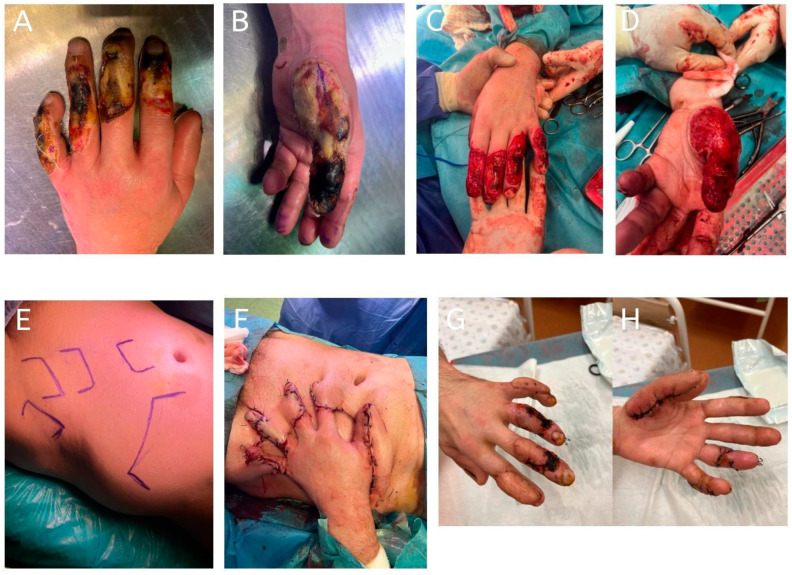
Case of a high-voltage electrical burn resulting in extensive tissue loss on the dorsal aspect of all five fingers. Coverage was achieved using five individual pedicled abdominal flaps, one for each finger. Flap division was performed 24 days postoperatively. The patient demonstrated good healing progress; however, 2 months after hospital discharge, the patient tragically died from an unrelated cause. (**A**,**B**): Initial presentation showing multiple dorsal defects on digits I–V; (**C**,**D**): appearance of the hand following radical debridement and preoperative preparation of the defect; (**E**): design of five individual pedicled abdominal flaps marked on the abdominal donor site; (**F**): insetting of the five pedicled abdominal flaps onto each of the five affected fingers; (**G**,**H**): postoperative appearance of the fingers 8 weeks after the procedure, showing satisfactory healing and coverage.

**Figure 3:**
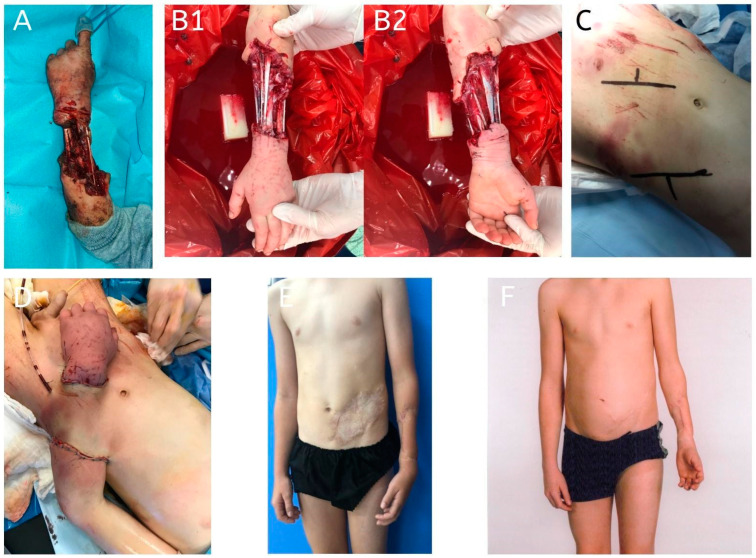
A 9-year-old patient presented with a severe circumferential forearm defect caused by a dog bite. Given the extent and nature of the tissue loss, a tunneled abdominal flap was performed. Postoperatively, a surgical site infection was observed but was successfully managed with regular local wound care. Flap division was performed 26 days after the procedure. Both the donor site and the palmar side of the forearm required coverage with a split-thickness skin graft. As part of the second stage of reconstruction, two tissue expanders were placed in the donor site area for 3 months. The final stage involved abdominoplasty, resulting in a satisfactory functional and aesthetic outcome. (**A**): Initial presentation of the patient on admission, showing a severe circumferential defect of the forearm; (**B1**,**B2**): appearance of the defect following preoperative debridement; (**C**): design of the tunneled abdominal flap marked on the abdominal donor site; (**D**): placement and insetting of the tunneled abdominal flap into the forearm defect; (**E**,**F**): final result before and after abdominoplasty, demonstrating functional and aesthetic restoration of the abdominal donor site.

**Figure 4:**
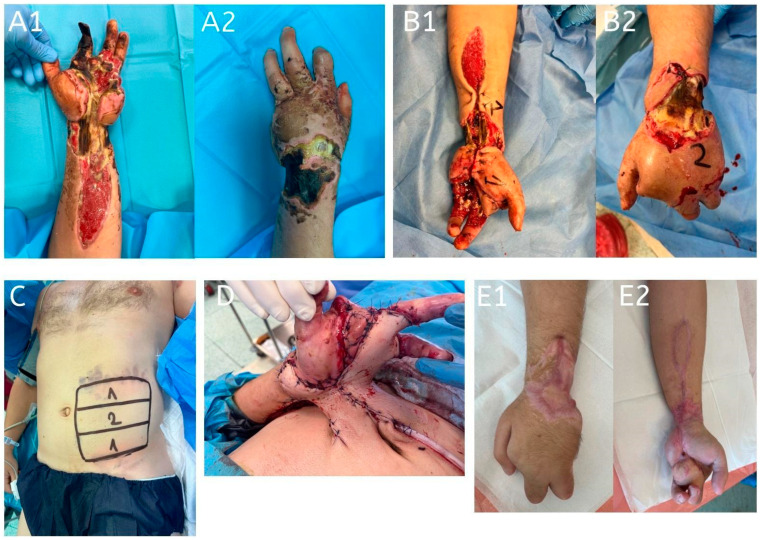
A 31-year-old patient sustained bilateral hand, wrist, and palmar forearm defects due to a high-voltage electrical burn. Reconstruction was performed using a three-pedicled abdominal flap in conjunction with a full-thickness skin graft on the palmar forearm and amputation of the second digit. The amputation was performed from the volar side, with the dorsal skin of the digit repurposed to cover the palmar defect partially. Postoperatively, partial necrosis was observed in one of the flaps, requiring surgical debridement, while a second flap experienced complete necrosis. The third flap healed successfully without complications. Flap division was performed 22 days after the initial procedure. (**A1**,**A2**): Initial presentation on admission, showing bilateral hand, wrist, and palmar forearm defects caused by a high-voltage electrical burn; (**B1**,**B2**): preoperative images with the anatomical sites marked for the design of the three pedicled abdominal flaps; (**C**): design of the three pedicled abdominal flaps on the abdominal donor site; (**D**): insetting of the three pedicled abdominal flaps to cover the bilateral hand, wrist, and forearm defects; (**E1**,**E2**): follow-up images 6 months postoperatively, showing the final result with stable soft tissue coverage and preserved hand function.

**Figure 5:**
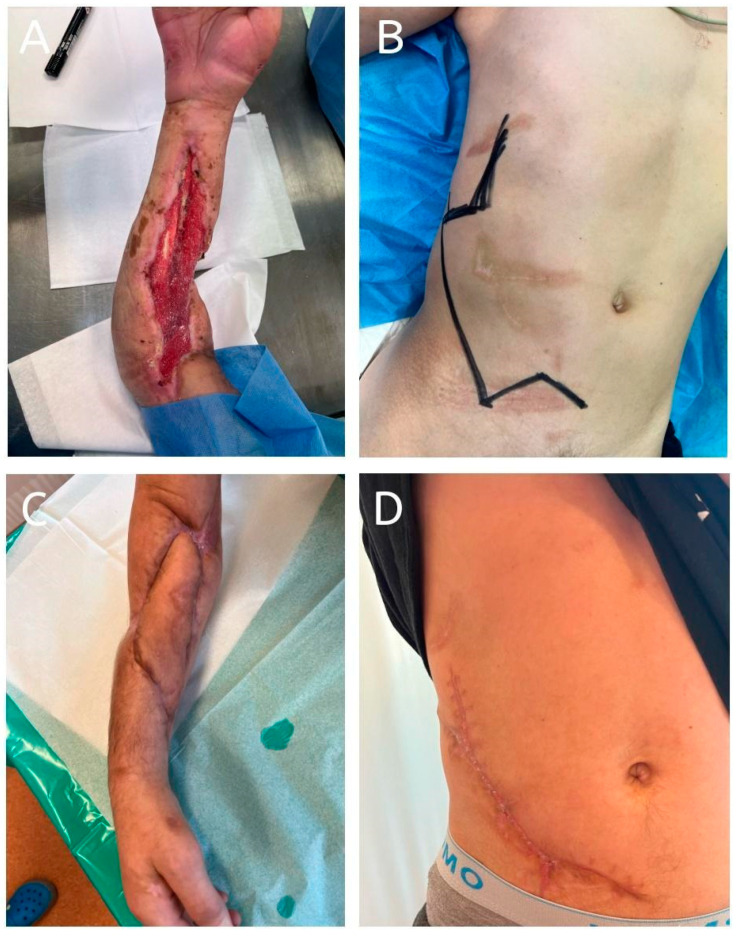
A 28-year-old patient presented with a soft tissue defect on the palmar side of the forearm following an agricultural injury. The injury resulted in the absence of both the radial and ulnar arteries, with exposure of the median nerve. Reconstruction was performed using a pedicled abdominal flap. No postoperative complications were observed, and flap division was carried out 22 days after the initial insetting. The patient achieved full healing with satisfactory functional and aesthetic outcomes. (**A**): Preoperative image showing the soft tissue defect on the palmar forearm with exposed median nerve; (**B**): design of the pedicled abdominal flap on the abdominal donor site; (**C**): healed flap after successful division and complete integration; (**D**): healed donor site 18 months postoperatively, demonstrating good aesthetic and functional results.

The nature and location of soft tissue loss varied among the patients, necessitating a highly individualized approach to flap design and selection. The characteristics of the tissue loss had a direct influence on the choice of flap configuration. The characteristics of the patients included in the present study are comprehensively presented in Table 1. Moreover, various cases included in this study are presented in Figure 1, Figure 2, Figure 3, Figure 4 and Figure 5.

Postoperative complications were observed in several patients, though all ultimately achieved stable soft tissue coverage without the need for additional free flap procedures or secondary reconstruction. Surgical site infections occurred in two patients, both of which were successfully managed with antibiotic therapy and local wound care. Marginal flap necrosis was noted in two patients, who were treated with routine dressing changes and surgical debridement. Moreover, partial flap necrosis was observed in two patients, one of whom underwent a procedure involving the use of three flaps. Of the three flaps, one exhibited partial necrosis, another demonstrated complete necrosis, and the third healed without complications. Apart from this case, another complete flap necrosis was reported. In this instance, the necrotic flap was repurposed as a “biological dressing”, promoting the formation of granulation tissue and facilitating wound closure with a split-thickness skin graft.

A single case of delayed bone union was observed in the patient who underwent a combined procedure involving a pedicled abdominal flap and a vascularized bone graft. Despite this delay, the union was eventually achieved without the need for further surgical intervention.

Long-term follow-up revealed no cases of postoperative finger stiffness. The flap design aimed to minimize patient immobilization and appropriate physiotherapy was provided both before and after flap division. Any observed limitations in finger range of motion were attributed to the initial tendon injury rather than postoperative complications. All patients maintained stable soft tissue coverage, and no long-term issues with wound healing were reported.

In the absence of complications and with stable patient conditions, hospital discharge occurred five to eight days post-surgery. Patients were subsequently readmitted for flap division, which was performed 21 to 28 days after the initial abdominal flap procedure. In one exceptional case involving complete flap necrosis, flap separation was performed on day 11. The necrotic flap served as a “biological dressing”, promoting granulation tissue formation beneath it. This granulation tissue facilitated defect coverage with a split-thickness skin graft 10 days later.

Due to the relatively thin nature of the free-style pedicled abdominal flaps, no additional debulking procedures were required. Donor site complications were minimal, with no significant adverse events reported. The only notable case involved a pediatric patient who required a split-thickness skin graft to close the donor site. To further improve the functional and aesthetic outcome, a tissue expander was used at the donor site, and the final stage of reconstruction included an abdominoplasty, resulting in a satisfactory functional and cosmetic result.

## 4. Discussion

Soft tissue defects of the hand and upper extremity, as seen in our cases of trauma and infection, require meticulous reconstruction to restore both function and appearance. Despite advancements in micro- and supermicrosurgery, the pedicled abdominal flap remains a valuable option. Its versatility and reliability make it particularly useful in complex cases where local or free flaps are unsuitable or when previous reconstructions have failed. In all our cases, the defects involved critical structures, such as tendons, bones, joints, or nerves, necessitating robust soft tissue coverage. As a result, primary treatment with a skin graft alone was not a viable option, highlighting the essential role of pedicled abdominal flaps in these challenging reconstructions.

Although free flaps are the favorable option in reconstructive procedures of the upper limb, various papers have demonstrated that in certain situations, pedicled abdominal and groin flaps may be a better option [3]. Al-Qattan et al. [3] discussed how the specific indications of utilizing these flaps may consist of reconstructions of complex defects in children under 2 years old, coverage of digital stump defects to facilitate future toe-to-hand transfers, management of high-voltage electrical burns where the hand remains viable due to collateral blood supply, salvage of the thumb ray in cases of high-voltage burns complicated by radial artery thrombosis, preservation of finger length following multiple digital amputations in manual workers, treatment of hand mutilation injuries, and addressing multiple soft tissue defects affecting the digits, hand, or forearm. We strongly support this rationale, as our clinical experience agrees with the logical and effective use of these flaps in such challenging cases. Their ability to provide reliable coverage, maintain vascular integrity, and support functional recovery highlights their continued importance in reconstructive strategies for upper limb defects.

Reconstructing upper extremity defects caused by high-voltage electrical burns and full-thickness burns presents a significant challenge due to the extensive injury to recipient vessels and surrounding soft tissue. Baumeister et al. [6] reported a 10% failure rate for free flaps in patients with full-thickness burns and a 19% failure rate in those with electrical injuries, with all failures occurring when the procedure was performed within six weeks post-injury. Similarly, other studies have highlighted that free flap loss rates in acute burn cases, approximately 10%, are notably higher compared to other reconstructive indications [7]. These findings underscore the critical need for timely and reliable coverage of upper extremity defects. Given the potential for endothelial damage in recipient vessels following burns, free flap procedures carry a significant risk of anastomotic thrombosis. Attempting this approach under such circumstances risks flap failure and jeopardizes the viability of the entire upper extremity, especially when its primary vascularization is already compromised. In these cases, we advocate for the use of pedicled abdominal flaps as a safer alternative.

Additionally, distorted hand anatomy may not result solely from the initial trauma, but can also arise from failed reconstruction attempts. In situations where perfusion is dependent on a single artery, such as after hand replantation, any compromise of the vascular supply may result in complete loss of the limb. Based on our experience, a pedicled abdominal flap is not only a safer option but, in many cases, the only feasible solution to ensure stable coverage and preserve hand function. Although there are no clear studies indicating a higher risk of free flap loss or failure in diabetic patients [8], microsurgical anastomosis in an inflammatory and purulent environment significantly increases the risk of flap loss [9]. These factors should be taken into consideration when operating on patients with defects associated with an infectious origin, such as necrotizing fasciitis of the upper limb.

The versatility of free-style pedicled abdominal flaps allows for the coverage of various tissue defects, depending on the surgeon’s creativity and technical skill. As demonstrated in Figure 2, multiple small abdominal flaps can be used to fit multiple areas of tissue loss, thereby eliminating the need for future de-syndactylization procedures and enabling greater hand mobility during the pre-division period [10]. When both dorsal and palmar structures are exposed, the use of double abdominal flaps is often the most rational and effective approach.

A significant advantage of free-style pedicled abdominal flaps is their customizability. The size and shape of the flap can be tailored to the specific dimensions and characteristics of the defect. Ideally, the pedicle should be long enough to facilitate significant hand and wrist mobility, as well as simplify dressing changes during the healing process. In addition, these flaps have demonstrated success in the treatment of complex circumferential hand defects [11,12], a finding consistent with our clinical experience.

Despite their versatility and effectiveness, pedicled abdominal flaps have several notable disadvantages. One of the most significant drawbacks is the need for a two-stage procedure, as the flap must be divided later [3]. This prolongs the overall treatment timeline and necessitates hospital readmission. Patient discomfort is another key limitation, as the hand must remain attached to the abdomen, often in a restrictive and uncomfortable position [13]. Subsequently, this leads to limited hand mobility and delayed initiation of physiotherapy, which are additional concerns. The immobilization required for flap adherence can lead to stiffness, particularly in uninjured digits, which may impair overall hand function [3]. Flap debulking may also be necessary in some cases, requiring further surgical intervention to achieve an optimal contour. Finally, in the context of acute trauma, the inability to elevate the hand postoperatively can increase swelling and delay rehabilitation [10].

When planning the use of pedicled abdominal flaps for upper limb reconstruction, careful consideration must also be given to donor site closure. Primary closure is preferred, as it minimizes donor site morbidity, reduces the risk of complications, and enhances the final aesthetic outcome. However, when the defect is too large for primary closure without excessive tension, the donor site should be covered with a skin graft. In such cases, a second-stage aesthetic correction, such as abdominoplasty, may be required to achieve a satisfactory functional and cosmetic result [4,5].

This study has several limitations. The small sample size of 20 patients may limit the generalizability of our findings to broader patient populations. Additionally, postoperative functional outcomes, such as hand mobility and dexterity, were not objectively measured using standardized assessment tools like the DASH (Disabilities of the Arm, Shoulder, and Hand) score, which could have provided a more comprehensive evaluation of hand function. Future studies with larger cohorts and the inclusion of objective outcome measures are needed to better assess the efficacy and functional impact of free-style pedicled abdominal flaps in upper extremity reconstruction.

## 5. Conclusions

Despite advancements in and the widespread use of microsurgical techniques, free-style pedicled abdominal flaps continue to hold a significant role in plastic and reconstructive surgery, particularly for the management of complex upper limb defects. Their simplicity, robust vascularity, and adaptability make them a valuable option in specific clinical scenarios where free flaps may be contraindicated or high-risk. This approach offers a reliable method of reconstruction, especially in patients with compromised health or in cases involving extensive tissue loss, electrical burns, or defects requiring staged reconstructions. The ability to customize the flap design to the needs of the defect, combined with favorable donor site characteristics, underscores its continued relevance in modern reconstructive surgery.

## Figures and Tables

**Table 1:** The characteristics of the patient sample.

Sex and Age (F—Female; M—Male)	Type of Abdominal/Groin Flap	Tissue Defect	Cause of the Defect	Time of Flap Division (Days)	Complications
M 82	Pedicled flap	Palmar hand defect	Machine injury	22	None
M 36	Pedicled flap	Degloving injury of volar hand involving four digits (2nd–5th)	Machine injury	23	None
M 69	Pedicled flap	Complete circumferential upper extremity defect	Necrotizing fasciitis	11	Complete flap necrosis
M 26	Pedicled flap	Palmar forearm defect	Agricultural injury	26	None
F 11	Pedicled flap	Dorsal wrist defect	Complication after surgery in another center	22	Partial flap necrosis
M 34	Pedicled flap	Palmar side of forearm defect	Agricultural injury	22	None
M 63	Pedicled flap	Dorsal hand, wrist, and forearm defect	Necrosis after injury	28	None
M 37	Pedicled flap	Palmar side of forearm defect	Agricultural injury	23	None
M 28	Pedicled flap	Palmar side of forearm defect	High-voltage electric burn	22	None
M 32	Pedicled flap	Palmar defect	Necrosis after injury	21	None
M 42	Pedicled flap	Palmar defect	Necrosis after industrial injury	24	None
M 34	Double pedicled flap	Coverage of degloved basal phalanges and metacarpophalangeal joints after unsuccessful metacarpal replantation in another center	Press machine	24	Marginal flap necrosis
M 54	Double pedicled flap	Partial hand-degloving injury	Agricultural injury	26	Complete necrosis of first flap and partial necrosis of second flap
M 31	Triple pedicled flap	Exposure of the dorsal side of the wrist, amputation of 2nd and 5th digits, exposure of the carpal tunnel and palmar side of the forearm	High-voltage electric burn	22	None
M 44	Five pedicled flaps for each digit	Dorsal side of all five digits	High-voltage electric burn	24	Surgical site infection
M 9	Tunneled abdominal flap	Forearm circumferential defect	Dog bite	26	None
M 32	Tunneled abdominal flap	Hand and wrist circumferential defect	Thermal burn	28	None
F 29	Tunneled abdominal flap	Degloving injury	Press machine	27	None
			data		
F 28	Tunneled abdominal flap	Hand and forearm degloving injury	Press machine	26	Surgical site infection
M 43	Pedicled flap with nonvascularized bone graft from the iliac	Dorsal hand defect with absence of 2nd to 4th metacarpal bones	Crush injury	27	Delayed union

## Data Availability

The data that support the findings of this study are available from the corresponding author, upon reasonable request.
